# The role of distance and transportation in decision making to seek emergency obstetric care among women of reproductive age in south–South Nigeria: A mixed methods study

**DOI:** 10.1002/ijgo.14103

**Published:** 2022-02-15

**Authors:** Mandu S. Ekpenyong, David Matheson, Laura Serrant

**Affiliations:** ^1^ Department of Nursing Manchester Metropolitan University Manchester UK; ^2^ Department of Education for Health, Faculty of Education, Health and Wellbeing University of Wolverhampton Walsall UK

**Keywords:** decision making, distance, Nigeria, role, transportation, women of reproductive age

## Abstract

**Objective:**

To explore the role of transportation in seeking emergency obstetric care among women with obstetric complications.

**Methods:**

A mixed‐methods design. The study population comprised women aged 15–49 years who had experience direct obstetric complications and were attending the health facility for care at the time of this study. A total of 318 women completed the questionnaires, and in‐depth interviews were held for six women who were purposively selected from the 318 respondents. Both questionnaire and semi‐structured interviews were used in collecting data for this study. Quantitative data were analyzed using SPSS using both inferential and bivariate analysis, and a qualitative content analysis was carried out on the qualitative data.

**Results:**

Of the 318 respondents, 290 (91.2%) accessed health facilities by motorized transport with 28 (8.8%) on foot. Mode of transportation was related to regularity at prenatal care (*P* = 0.003), with those who came on foot being regular attenders compared with those that came on motorized transport.

**Conclusion:**

An efficient and suitable transport system as well as distance are key factors influencing women’s role in decision making to seek care.

## Abstract 2

The aim of this study was explore the role of transportation in seeking emergency obstetric care among women with obstetric complications. To achieve this aim, a mixed method design (both quantitative and qualitative methods) was adopted. Our findings revealed that health care accessibility was influenced by mode of transportation to the facility. The influence was such that women who came on foot were more regular attenders compared with those that came on motorized transport. An efficient and suitable transport system and distance are key factors influencing women’s role in decision making to seek prenatal care.

## 1 INTRODUCTION

Transport and health are interlinked on many levels, with transport directly and indirectly influencing health, and health status influencing transport options. Transportation is an important barrier to accessing obstetric care for many pregnant and postpartum women in developing countries, particularly in rural areas.^1^ Transportation also facilitates the timely and reasonable delivery of basic health, education; it connects communities to trade and information with one another and can empower vulnerable groups. However, low‐income countries such as Nigeria are yet to completely accept and understand the role of transport in accessing maternal healthcare services and improving poor people’s health. Women in Nigeria are faced with long distances and significant transportation costs to access and use maternal healthcare services in health facilities. An effective transportation system plays an important role in responding to an emergency promptly and effectively.^2^

Most maternal deaths from direct causes (such as obstructed labor, eclampsia, infection, hemorrhage, or unsafe abortions) could be prevented if women received timely care at critical moments.^3^ Maternal mortality is a major public health problem, particularly in sub‐Saharan Africa, where half (50.4%) of all maternal deaths worldwide occur.^4^ In 2005, the maternal mortality ratio in sub‐Saharan Africa was estimated at 900 maternal deaths per 100 000 live births, which was by far the highest in the world.^5^ Most pregnancies of healthy mothers end with the birth of a live baby. However, in many cases, childbirth is not the joyous event it should be but a time of pain, fear, suffering, and even death.^6^

Transportation costs have been reported as one of the factors shown as having influence on maternity care access and utilization.^3^ Krasovec^7^ revealed that the Ministry of Health and the introduction of a two‐way radio system to link the hospital increased the number of women referred with major obstetric complications from 0.9 to 2.6 per month following the replacement of motorbikes for referral from primary health centers with a four‐wheel drive vehicle. Transport played some role in increasing women’s access to effective care, although some of these effects may have been an indirect result of additional women using the referral facility because of its upgraded status. Transport may have more greatly helped the survival of women coming from longer distances, while enhanced treatment at the hospital had a greater role in improved outcomes for women living closer to the health facility.^7^

The provision of care for women during pregnancy and childbirth is essential to ensure a healthy and successful outcome of pregnancy for the mother and her newborn. All pregnant women are at risk of obstetric complications. About 15% of pregnancies and childbirths need emergency obstetric care because of risks that are difficult to predict.^8^ Most life‐threatening complications occur during labor and delivery, and these cannot be predicted. Every pregnant woman needs access to facilities with capabilities to provide emergency obstetric care services.^8^ Emergency obstetric care is one of the important components of Safe Motherhood programmes; it refers to services provided in cases that threaten the lives of the mother and fetus, and that require emergency intervention during pregnancy and during or after childbirth.

Emergency obstetric care consists of the speedy evaluation of pregnant women on admission, and the early treatment in the event of the mother or infant showing life‐threatening signs or symptoms. Furthermore, after calming the situation, if needed, emergency obstetric care involves transferring the mother/infant to higher‐level health care and providing safe blood transfusion in healthcare facilities.^9^ Neither effective prenatal care nor identifying risk will help women if emergency obstetric care is not accessible, or not used.

This study set out to explore the role of transportation in seeking emergency obstetric care among women with obstetric complications. In this study, direct obstetric complication is defined as an acute condition arising from a direct cause of maternal death, such as antepartum or postpartum hemorrhage, obstructed labor, postpartum sepsis, complications of abortion, pre‐eclampsia or eclampsia, ectopic pregnancy, and ruptured uterus.^10^

## 2 MATERIALS AND METHODS

We used a mixed‐method design (quantitative and qualitative design) to assess the role of transportation and travel time in seeking emergency obstetric care. Combining quantitative and qualitative research approaches offers a strong potential for identifying and exploring factors affecting maternity care services utilization. We used a sequential mixed‐method design, where quantitative research was followed by qualitative research. Three‐hundred and eighteen women of reproductive age who had experienced direct obstetric complications and were attending one of the tertiary health facilities in Delta State Nigeria completed the questionnaires. Six in‐depth interviews were conducted for women who were purposively selected from the 318 quantitative respondents who completed the questionnaire survey. The women included were all consenting patients presenting with direct obstetric complications and were between 15 and 49 years of age.

The quantitative data were analyzed using SPSS version 20.00 (IBM, Armonk, NY, USA). Descriptive and inferential statistical methods were used to analyze the quantitative data. Bivariate analysis and Pearson’s χ^2^ test was used for categorical data. The level of significance was set at *P* value of 0.05 or less. Qualitative interviews were transcribed verbatim, and the lead author conducted content analysis with codes checked by a co‐author.

The emergency obstetric care provided in the health facility where this study was conducted included treatment for postpartum hemorrhage, obstructed labor, eclampsia, abnormal presentation of fetus, and miscarriage.

Patients and public were not involved in this study in any way.

On December 16, 2014 the present research received ethical approval from the University of Wolverhampton School of Health and Wellbeing Ethics Committee and on February 3, 2015, an approval from the Ethical Committee of the Tertiary Health Facility was obtained. Informed consent was obtained from the study participants.

## 3 RESULTS

### 3.1 Demographic characteristics of the women

The demographic data are essential to this research study. Table 1 shows the descriptive analysis of individual and household factors. The information on the sociodemographic characteristics of respondents reveals that the age range of the women was 15–45 years with a mean ± standard deviation age of 26.4 ± 5.4 years. Most (54.1%) respondents were within 26–35 years of age, and 46 (14.5%) were within the ages of 36–45 + years and above. Two hundred and fifteen (67.6%) respondents were in a monogamous union, while 46 (14.5%) were in a polygamous union. The occupational information of respondents showed that 102 (32.1%) were civil servants and the rest belonged to other occupations. Respondents' level of education ranged from primary to post‐secondary education 172 (54.1%). The distribution of respondents by educational qualification showed that half 163 (51.3%) of the respondents had completed post‐secondary education, and eight (2.5%) had no formal education.

**TABLE 1:** Demographic characteristics of the study respondents^a^

Characteristics	Frequency (*n*)	Percentage	Urban	Rural
Age at last birthday, years				
15–25	100	31.4	65 (65.0)	35 (35.0)
26–35	172	54.1	140 (81.4)	32 (18.6)
36–45+	46	14.5	39 (85.0)	7 (15.0)
Marital status				
Married	238	74.7	189 (79.4)	49 (20.6)
Single	64	20.1	42 (65.6)	22 (34.4)
Separated	8	2.5	4 (50.0)	4 (50.0)
Widowed	6	1.9	6 (100.0)	–
Divorced	2	0.6	2 (100.0)	–
Family type				
Monogamous	215	67.6	179 (83.3)	36 (16.7)
Single parenting	57	17.9	35 (61.4)	22 (38.6)
Polygamous	46	14.5	29 (63.0)	17 (37.0)
Occupation				
Civil servant	102	32.1	95 (93.1)	7 (6.9)
Traders	65	20.4	43 (66.2)	22 (33.8)
Unemployed	58	18.2	36 (62.1)	22 (37.9)
Student	51	16.0	39 (76.5)	12 (23.5)
Artisan	31	9.7	21 (67.7)	10 (32.3)
Multinational company	9	2.8	7 (77.8)	2 (22.2)
Clergy	2	0.6	2 (100.0)	–
Educational status				
Post‐secondary	163	51.3	148 (90.8)	15 (9.2)
Secondary education	92	28.9	70 (76.1)	22 (23.9)
Primary education	55	17.9	23 (41.8)	32 (58.2)
No formal education	8	2.5	2 (25.0)	6 (75.0)
Estimated monthly income				
Below minimum wage	179	74.3	154 (86.0)	25 (14.0)
Above minimum wage	62	25.7	40 (64.5)	22 (35.5)
Religion				
Christianity	270	84.9	215 (79.6)	55 (20.4)
Islam	44	13.8	26 (59.1)	18 (40.9)
Traditionalist	4	1.3	2 (50.00)	2 (50.0)
Ethnic group				
Igbo	130	40.9	95 (73.1)	35 (26.9)
Yoruba	49	15.4	43 (87.8)	6 (12.2)
Hausa	30	9.4	14 (46.7)	16 (53.3)
Others (Ibibio/Efik, urohobo/ijaws, Ishekiri/Ukwani	109	33.2	92 (84.4)	17 (15.6)

^a^ Values are given as number (percentage) unless otherwise stated.

The income distribution of respondents revealed that 179 (74.3%) respondents had income level above (₦18 000) national minimum wage per month; whereas 62 (25.7%) reported income level below (₦18 000) national minimum wage. Two‐hundred and seventy (84.9%) respondents were Christians, and the remainder belonged to other (Islam, traditionalist) religions. For ethnicity, 130 (40.9%) were Igbo and 30 (9.4%) were Hausa.

**FIGURE 1:**
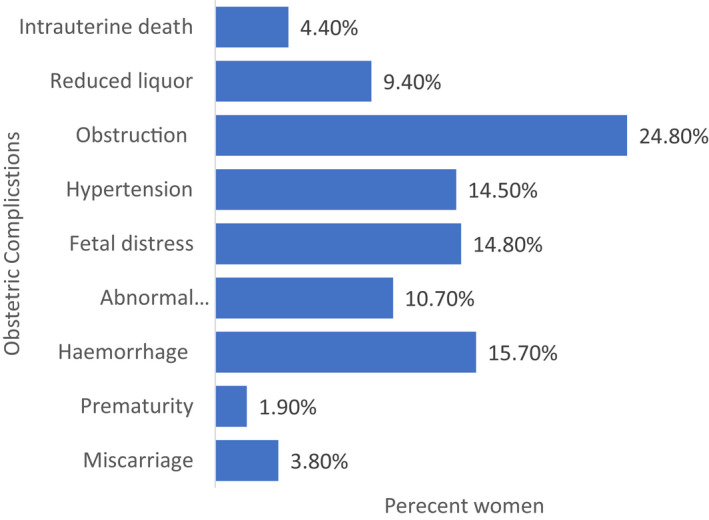
Obstetric complications among the study participants

Figure 1 illustrates that out of the nine known categories of signs for emergency obstetric complications during labor and childbirth, obstruction was the highest (24.8%), compared with prematurity (1.9%).

### 3.2 Means of transportation

Respondents’ means of transportation to the hospital shows that majority 291 (91.2%) of the respondents traveled to the health facility by motorised transport, while 8.8% representing 28 respondents came on‐foot. Suggesting that a good transport system put in place for pregnant women by the government will allow the women to utilise the health facility more for their needed healthcare.

### 3.3 Delays to getting to the hospital

One hundred and five (33.0%) respondents reported having difficulties associated with transportation. Respondents provided various reasons associated with difficulty in transportation, 77 (58.8%) 2 respondents claimed that too far a distance was the major difficulty associated with transportation, when compared with 4 (3.1%) respondents that reported high transportation cost as difficulty associated with transportation (Table 2).

**TABLE 2:** Distribution of women according to access to health facility factors

Characteristics	Frequency (*n*)	Percentage
Mode of transportation to health facility		
Motorized transport	290	91.2
On foot	28	8.8
Difficulty associated with transportation		
Yes	105	33.0
No	213	67.0
Kinds of difficulties associated with transportation		
Too far a distance	77	58.8
Lack of ready transportation	28	21.4
Traffic hold‐up	12	9.2
Problems of bad road	10	7.6
No transportation fare	4	3.1

### 3.4 Transportation, distance and time as a determining factor to health care utilization

Women face numerous problems in accessing appropriate maternity care during pregnancy and delivery. Women reported that limited or lack of public transport services and police stops make maternal healthcare service use difficult. The timing of the transport and distance to the facility added further difficulty and costs to the use of services, particularly during the emergency period at night. One of the aspects that affected the belief structure of Nigerian women remains access to reproductive healthcare services.

Closeness! The place is nearer to my house, and it takes such a little while to get to the hospital.—(P1).

Distance influences people’s decision making. The time spent in reaching a facility early is influenced by lack of readily available transport, police stopping drivers for bribes, location, and geographical distribution of these facilities. Once a decision to seek care has been made, other obstacles must be overcome in reaching the facility. In Nigeria, the modes of transportation mainly vary based on where you reside and who you are (in terms of socio‐economic status). In rural areas, delays due to distance and the unavailability of transportation are common.

It is a walkable distance from my house.—(P3).

Choice of health facility was made as a function of proximity with distance and time of service as disincentive factors to using a healthcare facility for skilled health care.

My inability to utilize the maternal health care services as much as I would have loved to was partly due to the distance. I get discouraged to come to seek care here because my house is too far from here and the timing for these services is also not favorable for me.—(P4).

Analysis of the narratives indicated that the logistics of the health systems of Nigeria have a lot of barriers in the form of transport services due to unavailability of an ambulance within the health systems to assist in moving patients from one facility to another.

One of the study participants has this to say:

I was referred to this hospital from a private clinic for delivery, but I was not provided with an ambulance or other form of transport services for my referral. My husband and I had to get a taxi from the private clinic where we were referred from to this facility and the cost was high. The situation was so discouraging.—(P1).

Transportation problems were reported to be worse at night due to bad roads, most drivers were not in operation during such hours, and this made transportation difficult during emergencies.

Most drivers do not want to operate at nights due to poor roads. Because of the drivers not operating at nights, people wait for many hours (even during emergency situations) before getting a vehicle and in most of the time the prices are very high as opposed the normal cost of fare in the area.—(P2).

The difficulties associated with transportation were increased by the lack of ready transportation/ambulance services, difficulty of traveling on bad roads, high cost of transportation fares to purchase prescribed medication or laboratory tests outside the health facility, too great a distance, and police demanding bribes from drivers.

Police should be banned from standing on the checkpoints because they are not useful standing on the roads than to collect bribe and delay people from getting to their destination on time.—(P5).

In the same vein, another participant reported that:

Because police usually demand for bribe, some of the drivers in turn double the transportation fare to make up for their loss.—(P6).

### 3.5 Socio‐economic factors and maternal decision to seek care

Table 3 shows socio‐economic factors associated with maternal decision to use maternity healthcare services. Results show that mode of transportation was related to regularity at prenatal care (*P* = 0.003), with those women who came on foot being regular attenders at prenatal care compared with those women how came by motorized transport. Travel time to the facility was significantly related to prenatal care attendance (*P* = 0.001), those who traveled for up to 30 minutes were more likely to report regularly to prenatal care compared with those who traveled for more than 30 minutes (Table 3).

**TABLE 3:** Association between socio‐economic factors and maternal decision to seek care

Characteristics	Attendance at prenatal care			Total	χ^2^	df	*P* value
	NR	OW	R				
Mode of transport							
On foot	2 (7.1)	5 (17.85)	21 (75.0)	28	16.645	3	0.003
Motorized	19 (6.78)	175 (62.5)	86 (30.7)	280			
Travel time, min							
≤30	4 (2.1)	70 (36.6)	117 (61.3	191	13.35	4	0.001
30–60	6 (8.2)	48 (65.8)	19 (26.0)	73			
>60	10. (20.0)	32 (64.0)	9 (18.0)	50			

Abbreviations: NR, not regular at prenatal care; OW, once in a while; R, regular at prenatal care.

### 3.6 Costs (medical/ transportation)

Costs due to lack of medical equipment to carry out the required tests were also mentioned by most of the participants in the in‐depth interview. Mothers were often asked to go to local chemists/laboratories/pharmacies to carry out prescribed tests and buy recommended medicines, which most of the time required the women going long distances.

On several occasions I was only given the simple medications like the multivitamins. The doctor asked me to go outside the health facility to get the major ones because the hospital did not have them. I had to go look for them outside to buy and these items are almost double the price outside the health facility. Items that are not available in the local pharmacies and chemist shops around are bought from other nearby towns, which sometimes it takes about an hour to travel there, and we pay so much on transportation to get to these places.—(P2)

The participants expressed concern about the poor state of their transportation system and how this has contributed to non‐use of skilled birth attendants. The side of the cost issue became clear during the interviews. Even though the cost of medical treatment might be a concern as well, the traveling costs, moving from one facility to another to buy medications and carry out laboratory tests often made the visit unaffordable for poor patients.

## 4 DISCUSSION

Distribution of respondents by age clearly shows that women continue childbearing until their late 40s. Most (238; 74.8%) of the respondents were married, while (1.9%) were single. Evidence found in the literature showed that marriage is essential in the formation of family and maternal health.^4^, ^11^ The implication of the marital status distribution is that women still found themselves taking care of their pregnancy or children alone as single mothers, separated women, divorcees, or widows. This situation increased the possibility of not having good health care because most women may not have the economic power to survive alone.

This study demonstrates that mode of transport and travel time were the major determinants in making the decision to seek maternity care in health facilities. This might have been a result of the difficulty associated with transportation and the high cost of transportation, as most of the single women in this study were either students or unemployed. The findings of this study also reveal that cost, mode of transport, and difficulty associated with transportation were the major factors influencing women’s decision to seek maternity care. The lower (≤30 min) the travel time, the more likely it was for the women to make a good decision to seek care. More of the women who visited the health facility on foot (75%) were more regular at prenatal care. The women in the present study bear the cost of transportation during visits to a health facility; and this comes when the mode of transport being used is commercial and should be subsidized by the government to encourage them to continue using health facilities for their medical needs. Conversely, Shehu et al.^12^ stated that transport‐related costs could be a primary factor in deterring patients from seeking treatment even when a vehicle can be obtained, cost can be prohibitive and not even related to distance.

The present study also revealed that women who visited the health facility on foot were more regular attenders at prenatal care. This again might have been due to the closeness of the respondents' place of residence to the nearest health facility. Rose et al.^13^ found that the primary mode of transportation for women in labor remains walking, and care‐seeking practices often reflect the fear of delivery en‐route, the physical hardships of traveling in such a state, and cultural practices. The distance being too far was the strongest difficulty associated with transportation, followed by lack of ready transportation, lack of transport fare, bad roads, traffic hold‐ups, and police stops. Communities can assist in a number of ways in facilitating the transportation of women with emergency cases to health facilities. Similarly, it has been reported that excessive travel time and distance can influence patients in not seeking care at a health institutions and can also be a contributing factor to why women choose to deliver at home rather than at a health facility.^8^, ^14^ In line with this, Borghi et al.^15^ found that the average time it took women to travel to a health facility for care was 2.8 h, increasing significantly in the more mountainous regions. From the findings of the current study, it is recognized that the road network is not in a suitable condition for maternal and other health needs of communities who might be seeking care in the health facility.

In conclusion, the present study suggested that the respondents' efficient, reasonable, and suitable means of transport plays a key role in empowering pregnant/nursing mothers in decision making to seek the desired health care. The strongest difficulty associated with transportation was lack of transportation service, and regular attendance at prenatal care was higher (92.9%) among women who visited the health facility on foot. Emergency access to obstetric care is crucial because many pregnancy‐related complications are unpredictable, and many pregnant women spend a disproportionate time trying to get to a health facility with the ability to treat obstetric complications. As a result, public transportation and on foot remain the major modes of transportation for pregnant women, thereby cruelly limiting their capacity to reach needed care. To reduce the barriers that women face while accessing maternity care, governments and non‐governmental organizations should work with communities to identify barriers to transport and the most appropriate ways to dismantle these barriers. Building and improving the existing transportation system by the government to meet the needs of women who desire to use health facilities for care is desirable.

## CONFLICT OF INTEREST

There is no conflict of interest.

## AUTHOR CONTRIBUTIONS

EMS and SL conceived and designed the study; EMS and DM prepared the manuscript; and LS and DM supervised the study and contributed to paper editing.

## Data Availability

Research data are not shared.
